# CRISPR/Cas9-gene editing approaches in plant breeding

**DOI:** 10.1080/21645698.2023.2256930

**Published:** 2023-09-19

**Authors:** Himanshu Saini, Rajneesh Thakur, Rubina Gill, Kalpana Tyagi, Manika Goswami

**Affiliations:** a School of Applied Natural Science, Adama Science and Technology University, Adama, Ethiopia; b School of Agriculture, Forestry & Fisheries, Himgiri Zee University, Dehradun, Uttarakhand, India; c Department of Plant Pathology, Dr Yashwant Singh Parmar University of Horticulture and Forestry, Nauni, Solan, Himachal Pradesh, India; d Department of Agronomy, School of Agriculture, Lovely professional university, Phagwara, Punjab, India; e Division of Genetics and Tree Improvement, Forest Research Institute, Dehradun, Uttarakhand, India; f Department of Fruit Science, Dr Yashwant Singh Parmar University of Horticulture and Forestry, Nauni, Solan, Himachal Pradesh, India

**Keywords:** Agriculture, Crispr/Cas9, gene editing, genome, plant breeding

## Abstract

CRISPR/Cas9 gene editing system is recently developed robust genome editing technology for accelerating plant breeding. Various modifications of this editing system have been established for adaptability in plant varieties as well as for its improved efficiency and portability. This review provides an in-depth look at the various strategies for synthesizing gRNAs for efficient delivery in plant cells, including chemical synthesis and *in vitro* transcription. It also covers traditional analytical tools and emerging developments in detection methods to analyze CRISPR/Cas9 mediated mutation in plant breeding. Additionally, the review outlines the various analytical tools which are used to detect and analyze CRISPR/Cas9 mediated mutations, such as next-generation sequencing, restriction enzyme analysis, and southern blotting. Finally, the review discusses emerging detection methods, including digital PCR and qPCR. Hence, CRISPR/Cas9 has great potential for transforming agriculture and opening avenues for new advancements in the system for gene editing in plants.

## 1. Introduction/Basic Gene Editing Strategy

Various crop genomes have been modified by alteration of foreign genes of high plant breeding values to overcome the problems associated with conventional breeding approaches over the last two decades. New gene editing approaches or strategies are required to improve crop yield, quality, and resistance against various stresses in crop plants. The pros and cons of various genetically modified crops are already discussed in other reviews.^1–3^ Gene editing approaches can play a crucial role to accelerate crop improvement programs with genetic changes in crop genomes.^4–7^ Gene editing with site-specific nucleases introduces DNA double strand breaks (DSBs) at a target site to evoke DNA repair mechanisms and converted into genetic modifications such as gene replacement, gene insertion, and targeted mutagenesis. Non-homologous end joining (NHEJ) is the most widely used DSB repair mechanism in crop plants.^8,9^

CRISPR/Cas system is the recently developed robust genome editing technology influenced by the bacterial adaptive immunity against bacteriophages. In 2012, two groups lead by Jennifer A. Doudna from University of California, Berkeley and Emmanuelle Charpentier from Umea plant science center (UPSC), Sweden^10^ first time reported that a DNA endonuclease (monomeric), known as Cas9, from *Streptococcus pyogenes* can be easily mechanized to cut the double-stranded DNA at a specific site of genomic sequence with the help of complementary base pairing of a single-guide RNA (sgRNA). Genome editing in eukaryotes was studied and later by these studies, a single construct of Cas9 nuclease and designed sgRNA required for the process of transformation. After this breakthrough technology, CRISPR/Cas9 technique widely used for genome editing in various organisms including plants^11–13^ and human cells.^14,15^ After understanding the importance of CRISPR/Cas9 technique, several reports and reviews were published.

CRISPR-based genome editing has revolutionized the practice of plant breeding by providing a more precise, cost-efficient, and rapid tool for creating desirable traits in plants. This technology has enabled the transfer of beneficial traits from one species to another, while minimizing or eliminating undesirable traits.^16,17^ This system has also been used to develop new varieties of crops that are more resistant to diseases and^18^ environmental stress,^19^ and that has improved nutritional profiles.^20^ The model species *Arabidopsis thaliana* and *Nicotiana benthamiana* have been used in many studies investigating the effect of CRISPR-based genome editing in plants.^21,22^ These species provide an ideal platform for studying the effects of gene editing on plant physiology, as they are small, easily manipulated, and have fully sequenced genomes. By using *Arabidopsis thaliana* and *Nicotiana benthamiana* as model species in these studies,^13,23–26^ scientists are able to gain valuable insights into how CRISPR-based genome editing can be used to improve crop production.

In addition, CRISPR technology is now being employed in forest tree species, such as poplar,^27^ pine,^28^ and spruce^29^ to identify and develop gene-editing tools to modify the genetic makeup of trees. These tools have potential to improve the health and growth of trees by reducing their susceptibility to diseases, and altering the wood structure to create more valuable timber. Furthermore, researchers are exploring new ways to use CRISPR to introduce new traits that reduce the need of pesticides, increase wood production, improve wood quality, and increase the sustainability of forestry operations.^30,31^ Here this review will focus on the acceleration of plant breeding through gene editing strategy of CRISPR/Cas9 RNA-guided endonuclease (RGEN) system and how it is helpful to accelerate molecular plant breeding for crop improvement program.

## 2. Design and Synthesis of Target-Specific Guide RNAs

CRISPR/Cas9 (clustered regularly interspaced short palindromic repeats-associated protein 9) is a complexed, two-component system using a short guide RNA (gRNA) sequence to direct the Cas9 endonuclease to the target site. Modifying the gRNA independent of the Cas9 protein confers ease and flexibility to improve the CRISPR/Cas9 system as a genome-editing tool. The main objectives of CRISPR systems are improved biofortification, stress tolerance, and yield efficiency in diverse plants under both biotic and abiotic circumstances. CRISPR/Cas was once believed to be a defense mechanism used by bacteria to fend off viruses. Technology for genome editing is constantly being improved to reach higher standards of precision and accuracy. CRISPR/Cas system is based on innate immune systems seen in prokaryotes and archaea. The method is based on an enzyme (CAS) designed to fragment a single individual DNA strand and introduce mutations that damage a gene’s open reading frame. Now that the fundamental components of this system have been explored and developed for genome editing. A CRISPR RNA (crRNA), Trans-activating CRISPR RNA (TracrRNA), and the Cas9 nuclease are the essential components.

**Figure 1.**
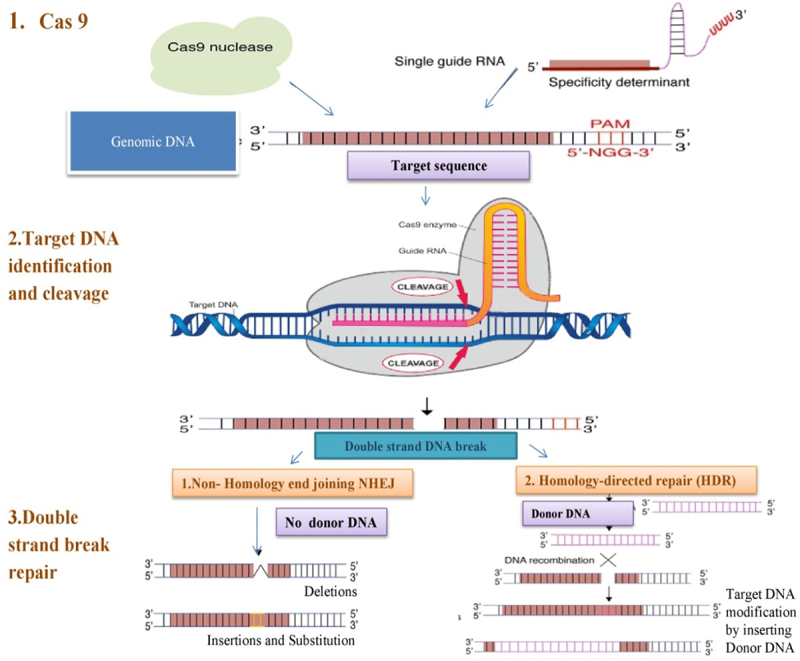
Overview of CRISPR/Cas9 technology for plant genome editing (i) two mechanisms for gene alteration include homology-directed repair (HDR) as well as non-homologous end joining (NHEJ) (ii) targeted DNA is cleaved and then repaired by NHEJ or HDR.

CRISPR/Cas systems can be designed by inserting the DNA target protospacer sequence into the crRNAs or sgRNAs. The editing potential of these tools has increased as a result of the discovery of several PAM (protospacer adjacent motif) specific Cas orthologs & polymorphisms.^32^ With this technique, foreign nucleic acids are specifically interfered with based on the sequence of short guide RNAs. Target locus needs alteration of genome *via* CRISPR/Cas9. DSBs, which happen when two repair processes alter the same gene, are brought on by the site-specific nucleases. Genes are deleted or fused using NHEJ, or non-homologous ending combining, is carried out without donor DNA. By using homologous portions as its foundation, homology-directed repair (HDR) adjusts gene sequences in response to even the smallest changes in either DNA strand (Figure 1).

Plants with heritable genome changes, such as (1) point mutations, (2) minute, arbitrary insertions or deletions (indels), (3) DNA fragment implantations, (4) DNA fragment reductions, and (5) chromosomal rearrangements that are specifically targeted, have been created using genome editing. Prior to genome modification with CRISPR/Cas9, a sgRNA must be designed that targets the desired gene. The fundamental component of the RNP compound is the ribonucleoprotein (CRISPR/Cas9) complex, which is composed of the gRNA and Cas9 nuclease. On the 3’ends of DNA targets, 5’-NGG-3’ sequences bearing the PAM motif are necessary.

The targeting sequence (crRNA), which is situated 20 nucleotides before the PAM sequence, will be divided into roughly three bases by the Cas9 nuclease. The target region’s gRNA can only attach to the genomic DNA if it has a particular protospacer neighboring motif (PAM). Later, the Cas9 nuclease separates the DNA into two strands (denoted by the scissors). A customized sgRNA with a Cas9 nuclease-recruiting domain and an aiming sequence (crRNA sequence) is required by the CRISPR/Cas9 system (tracrRNA).

The 20-nucleotide crRNA is the customizable component, which is complementary to the target DNA region in your target gene of interest, controls the Cas9 nuclease activity. Numerous online tools, such as CHOPCHOP or CRISPR Design, can find PAM sequences and list potential crRNA sequences within a particular DNA region. The most accurate crRNA for your research application can be chosen thanks to these algorithms’ predictions of impacts that are off-target across the genome. Ranking the risk of off-target impacts in its place, according to the amount and placement of mismatches in relation to the guide sequence, further user-defined incentives are applied by CasFinder^33^ and E-CRISP.^34^ DNA DSBs, which nucleases are optimized for causing, are caused by certain sequences (SSNs). The generated DSBs are then repaired by cellular DNA repair methods such NHEJ & homology-directed repair (HDR).

Because of erroneous sequence insertions or deletions, the break site primarily changes during DSB repairing NHEJ (indels). While HDR correction is the opposite, enables the exact insertion of predetermined sequences sent by a donor DNA template. When if a DNA template is present, natural repair mechanisms brought on by the double-strand break may cause a frame shift mutation that results in the knock-in or deletion of a desired sequence. The steps for design and synthesis of Target-specific guide RNAs into plant cells are as below:

### 2.1. Selection of the Target Gene

Mostly in CRISPR/Cas9 system, the combination produced by the tracrRNA and crRNA attracts Cas9 and gives instructions to cleave the DNA sequence at a particular genomic region. When the complex of tracrRNA and crRNA breaks down, sgRNA, which has just one strand of RNA, produces. The Cas protein is guided by the sgRNA and recognizes a conserved sequence. It precisely recognizes and binds to the protospacer neighboring motif after unraveling the DNA with two strands (PAM). The desired sequence and the matching crRNA sequence are linked upstream of the PAM.

### 2.2. Utilize the Online Tools to Design and Synthesize the sgRNA and Avoid off Target Activity

Numerous software programs can identify PAM sequences and provide a list of potential crRNA sequences in a certain DNA region, including on assessing sgRNA on-target efficacy, researchers have used E-CRISP,^34^ CHOPCHOP,^35–37^ and CRISPOR.^38^ These methods allow to select the crRNA that is most targeted by anticipating off-target consequences elsewhere within the sequence.^39^

### 2.3. Selecting the Appropriate Tool for Creating Guide RNAs and Web-Based Tools

Sequence-specific RNA (sgRNA) that specifies an appropriate genome can initiate CRISPR/Cas9 genetic manipulation in the desired gene.^40^ The greatest sgRNA design is essential for fruitful gene editing research because sgRNAs are exclusively accountable for delivering Cas9 to particular genomic locations. Numerous web-based tools for sgRNA design are available, each with an own set of capabilities and advantages.

Users typically need to specify a species, and every transgene is represented by a nucleotide sequence, a genetic region, or a domain designation when using web-based sgRNA synthesis tools. For every supply, a list of likely guiding nucleotides and related anticipated off-target locations is produced by a tool-specific algorithm.^41^ The majority of tools, albeit employing a range of techniques, strive to offer guide sequences that reduce the possibility of off-target impacts.^42^ Chop Chop, for instance, calculates efficiency scores using empirical data from a variety current study of Doench et al.^43^ According to the quantity and position of inconsistencies in relation to the guide sequence, Cas Finder^33^ and E-CRISP^34^ add particular user-defined consequences in which the probability of off-target impacts.

### 2.4. Applications-Specific Tools

Several specialized uses have spurred the creation of tools for designing sgRNA. The only technology that is now accessible, CRISPR-ERA,^44^ concentrates on applications in fly, beetle, and worm species, particularly some well model organisms *Caenorhabditis elegans* & *Drosophila melanogaster*. Applications in worm species are the main focus of Fly CRISPR.^45^ The only method for producing potential sgRNAs that operate with a range of nucleases, such as Cpf1 and *Staphylococcus aureus* Cas9, is the design tool on the benchling website.^46,47^ As a result of the unique characteristics that each instrument possesses using a number is recommended for strategies and pick guide sequences that are constantly anticipated to function correctly.

### 2.5. Assembling the sgRNA and Cas9 Protein Cloning of the Construct in Suitable Plant Binary Vector

A sgRNA is created by the base complementation and pairing of the crRNA and tracrRNA, mature products produced from the CRISPR locus. The sequence upstream of the PAM and the sgRNA pair causes a DSB. The two techniques for fixing the DSBs are HDR and NHEJ. During the NHEJ repair procedure, little parts could unintentionally be added or removed at the break site, altering the gene. When donor DNA is present, HDR precisely inserts or replaces nucleotides to fix the break spot.^48^

Homology directed repair is a frequent method for adding extra information to DNA for a particular alteration, like the addition of a fluorescent tag or the introduction of a certain mutation (HDR). Additionally, a DNA template from outside the body is needed. While single cell cloning and subsequent screenings for useful modifications are often required for HDR, it is a relatively ineffective approach. This is a protracted process that should not be undertaken carelessly. Actually, two rounds of single cell cloning are required to properly hit the gold standard. To show that the targeted change and not a passenger variant contained with the single cell clone produced the phenotype, the modification should be put back to its original state as a control (although this is rarely done).

## 3. Delivery of Guide RNAs in Plant Cells: Delivering the Plasmid Construct in to the Plant Through Different Transformation Techniques

To successfully alter the genome of plants, a variety of delivery techniques, most often utilized techniques include PEG-mediated protoplast, bombardment or biolistic method, floral-dip, as well as agrobacterium-mediated. Cas9 and gRNAs are commonly delivered into plant cells by physical methods like PEG-mediated protoplast transformation or biolistic callus transformation or agrobacterium-mediated T-DNA transformation. Gene targeting frequencies can be significantly increased by one to two orders of magnitude via geminiviral DNA replicon when compared to conventional *Agrobacterium tumefaciens* T-DNA transformation. Recent research has shown that the cytoplasmic replicating RNA virus tobacco rattle (TRV) is capable of transferring gRNAs into transgenic *Nicotiana benthamiana*, which expresses the Cas9 gene, to perform systemic genome editing, which is detectable in even two progeny plants. A Cas9-based approach based on DNA viruses for systemic genome editing in plants is not available at this time.

### 3.1. Transformation of the Genetic Code by Agrobacterium

The main technique for introducing gene-editing agents, such as base-editing tools, prime editing, and CRISPR/Cas variations agents, into plants is still agrobacterium-mediated genetic transformation.^49^ This procedure involves adding agrobacterium to explants that have T-DNA gene-editing cassettes integrated into them. Cells infected with the T-DNA containing the CRISPR cassette is probably to cause a stable genetic alteration in the host plant. Transgene-free gene editing is now possible because of the temporary creation of CRISPR tools through regenerative activities instead of using selecting.^50^

### 3.2. Crops Can Receive CRISPR Reagents via Biolistic-Based Distribution of DNA, RNA or Proteins

To strengthen crops resistance to *Agrobacterium* infection, common solutions include biolistic or particle bombardment. The membranes and cell wall of plants are physically penetrated using micro-projectiles made of protein-coated gold or tungsten that have been accelerated to extraordinarily high speeds. Only a handful of the numerous cargo types that can be transported by biolistic include ribonucleic proteins (RNPs) made of recombinant proteins, RNA, ssDNA, plasmid DNA, and IVTs. Important biolistic delivery restrictions include the time-consuming manufacturing of explants like callus or immature embryos with the potential for regeneration as well as the randomly integrating of cargo at various genomic loci when supplied as DNA.

### 3.3. CRISPR/Cas9 Vector Delivery Through PEG

A technique for genetic modification is employed when polyethylene glycol is present (PEG). The plasmid carrying gRNA & Cas9 is used to treat the protoplast when PEG is present. In this study, the promoter for gRNA and Cas9 are U3 & CaMV35S, accordingly, were used to introduce the first CRISPR constructs into maize.^51^

### 3.4. Pollen Magnetofection-Mediated Delivery

In the “magnetofection” approach of genetic modification, magnetic forces are employed to facilitate a vector’s absorption by magnetic nanoparticles (MNP). Currently, CRISPR/Cas9 vectors as well as the system’s vector/DNA less variants are used the most frequently for disseminating CRISPR/Cas9 components. Non-transgenic crops can be produced by using magnetofection and DNA-free editing. Cas9 mRNA and sgRNA transcription *in vitro* are used as the two methods for achieving this. Cas9 and MNP-coated gRNA are then employed and delivered to the protoplastor stigma. Cotton has profited from using this approach.^52,53^

### 3.5. Nanoparticle-Mediated Delivery

There are many different types of nanoparticles, such as mesoporous silica nanoparticles, carbon nanotubes, quantum dots, and metal/metal oxide NPs can be directly absorbed.^54–56^ According to^57–59^ a variety of crops, including maize, can be successfully genetically modified using silicon carbide whiskers; transgene-free plants can be made using CRISPR/Cas9 technology in other crops too like rice^60^ and cotton.^61^ It is possible to distribute the necessary nanoparticles and Cas9/gRNA ribonucleoproteins into newly developed tissues. To alter various pathways, multiple gRNAs must be included into a single plant transformation vector together with the proper promoters and terminators. It will be challenging to include a construct or numerous gRNAs because of the size of plant cells. Therefore, the employment of nanoparticles with many non-transgenic editing techniques, polycistronic tRNA-gRNA or polycistronic Csy4-gRNA will be useful. Effectiveness of any distribution strategy depends on both the selected approach and successful regeneration into complete plants.

### 3.6. Method Using a Pollen Tube or Floral Dip

In the past, pollen and plasmids were either mixed before being placed into the receptive stigma or plasmids were directly administered to the stigma’s surface.^62^ *Agrobacterium* solution was applied to wounded flowers with male and female parts before dipping them in it for effective gene transfer. The plant’s stage is essential for a smooth transition from vegetative to flowering state.

### 3.7. Bombardment-Mediated Delivery

Bombardment with a vector or Cas9/gRNA to deliver ribonucleoproteins. A “gene gun” or “biolistic gun” is required to carry out this transformation or gene transfer.^63^ The most common materials used as carriers for vectors or Cas9/gRNA ribonucleoproteins are gold, silver, and tungsten particles. By applying intense pressure, coated particles allow CRISPR/Cas9 components to flow through and enter explants. For this method, the explant type, helium pressure, particle size, and objective distance must all be optimized. On regeneration media, the modified explants are grown again under the proper selection pressure. There have been reports of successful Cas9/gRNA ribonucleoprotein delivery in maize, potatoes, and brassicas, followed by the regeneration of mutants.^64–66^

## 4. Detection

Following the successful delivery of gRNA into plant cells, detection methods are required to ensure the presence of intended mutations, measure indel efficiencies, isolate transformants, and to eliminate the CRISPR/Cas9 construct throughout the entire breeding process. To attain these goals, numerous analytical techniques and tools have already been used, all of which rely on prior knowledge of the sequences and genomes. CRISPR-mutant screening would be simple in the early phases of the genome editing process, but it becomes increasingly tricky as breeding populations are produced. As a result, choosing the best strategy among alternatives becomes essential. However, selecting appropriate detection methods also depends on the used for gRNA administration into plant cells.

### 4.1. Traditional Analytical Tools and Emerging Developments in Detection Methods to Analyze CRISPR/Cas9 Mediated Mutation in Plant Breeding

Since the inception of CRISPR/Cas9 system, HDR–mediated genome editing is being used to detect the point mutations in the edited plant.^67^ Despite their utility, its applications confront fundamental issues such as occasional occurrence and low efficiency of HDR, the inability to convert one base into another, and the failure of biallelic targeting.^68^ Conversely, allele-specific tailoring by CRISPR/Cas9 has evolved as a potential, cutting-edge method for addressing plants, biotic and abiotic stress concern.^69^ In this technique, gRNAs are used to discriminate single-nucleotide polymorphism differences. This method is employed to generate the base editing to expand the ability to convert unintended insertions and deletions of nucleotide into the wild-type nucleotide.^70–72^ To detect indels, insertion or deletion sites of many mutagenesis and SNPs from CRISPR/Cas9 driven mutant population, several conventional quantitative approaches such as sequencing (WGS, SS, and NGS), RT-qPCR, digital PCR, and endpoint fluorescence PCR have been extensively studied. Next-generation sequencing (NGS) based approaches like whole genome sequencing (WGS) and southern blot is used for the analysis of off targeting effects in plant breeding.^73,74^ In addition, Zhang et al.^52^ reported sanger sequencing (SS) as a suitable approach for Agrobacterium-mediated transformation. Furthermore, NGS data processing necessitates using a relevant and effective technology that generates accurate results to acquire CRISPR/Cas9-based genome editing outcomes. Thus, various high-throughput bioinformatics tools have been developed like CRISPR-DAV,^75^ CRISPR-GA,^76^ BATCH-GE,^77^ CRISPResso,^78^ CAS-analyzer,^79^ and CRISPR Match.^80^ Meanwhile, PCR assay-based approaches such as Kompetitative allele-specific PCR (KSAP), annealing at critical temperature PCR (ACT-PCR), allele-specific oligonucleotide PCR (ASO), and restriction fragment length polymorphism PCR (RFLP-PCR) have also been reported in number of known plant species.^81^ However, many of the approaches mentioned above have limitations, and as they are time consuming and tedious, these cannot be used as a frontline testing tactic. To circumvent these troubleshooting, various revolutionized changes may have been made in conventional plant breeding methods over time that has an economic importance and save time to increase the speed of breeding that can usually take up to 10 years for variety development.^82^

### 4.2. Web-Based Tools to Enhance the CRISPR/Cas9 Genome Editing Efficiency in Plant Breeding

Currently, widely used web-based tool for analyzing the NGS data that can be most preferable for base editing are CRIS.PY,^83^ SNP-CRISPR^84^), DeepBaseEditor,^85^ Be-Hive,^86^ BE-Designer,^87^ BEtarget,^88^ and FLASH-NGS.^89^ Along with this, a significant evolution has also happened in PCR approaches. Li et al.^90^ used a combination quantitative RT-PCR and high-resolution melting (qPCR-HRM) assay to find CRISPR/Cas9-induced mutations in rice plants. This approach is more empathetic and low-cost than other conventional PCR methods. Kalendar et al.^91^ used a KASP-modified method named allele-specific quantitative PCR (ASQ) to detect bi-allelic mutation by SNPs and indel mutation. The polycistronic tRNA-gRNA CRISPR/Cas9 (PGT/Cas9) system technology has also been implemented in *Arabidopsis* to identify the amorphic mutants in three generations using a straight forward PCR approach.^92^ A droplet digital PCR (ddPCR) is another most studied and effective technology for evaluating and detecting gene-editing frequencies in a variety of plant science field.^22,93–95^

Furthermore, the fast removal of transgenes and identification of transgene-free progenies from modified plants is crucial and a major concern for molecular breeders.^96^ A precise assessment of genetic heredity is still challenging since the CRISPR/Cas9 construct is still present in the plant cell after alteration, making it tough to separate the conveyance of induced mutations from offspring to succeeding generations. Thus, for the avoidance of off-target mutation effects, the maintenance of phenotypic stability, and the measurement of heredity, developing an effective and simple-to-implement technology is essential for plant improvement. In light of this, numerous approaches for obtaining transgene-free changed genomes have been developed, including fluorescence marker-assisted selection,^97,98^ active, programmed self-elimination system,^99^ H_2_O_2_-based leaf painting assay,^100^ TECCDNA-based genome-editing system,^101^ bolting-assisted selection,^102^ and moreover, these new tools enhance the CRISPR genome editing efficiency in plant breeding, which should pique the industry’s interest.

## 5. Analysis of Gene Editing Efficiencies

In comparison to other existing plant genome editing technologies like ZFNs and TALENs, the CRISPR/Cas9 tool has established its reputation as a versatile and adaptable alternative strategy and has made astonishing progress in the intervening years. Nonetheless, it has drawbacks, like gRNA delivery, low efficiency, off-target effects, and PAM requirements.^103^ For instance, various methods have been widely employed to maximize the mutagenesis efficiency. The efficacy of the classic CRISPR/Cas9 RGEN system demonstrated progressive off-target enhancement due to mutant inheritance in several plant species.^65,104^ However, because of their stringent PAM dependency, enormous size for essential transport and constrained gene target site efficiency brought on by blunt DSBs conferring low genome-wide specificity. So far, a versatile CRISPR/Cas class 2, Type-V system has been identified as a substitute.^105^ In this multifunctionality system, the Cas effector protein such as Cpf1, C2c1, C2c3, CasY, and CasX can interact with dsDNA, ssDNA, and ssRNA substrate type, which makes it an intriguing alternative for CRISPR/SpCas9 in plant genome engineering.^106^ After *Streptococcus pyogenes* Cas9 (SpCas9), *Lachnospiraceae bacterium* Cas12a (LbCas12a) has received the most attention and has been demonstrated to be efficient in a variety of plant species. Most recently, a combined system approach LbCas12a-ABE and Iterative Testing of Editing Reagents (ITER) increased 10–80% indel frequencies in wheat and maize plants.^107^ Additionally, two new coding sequences, ttHsCas12a and ttAtCas12a+int, were discovered, helping further to boost the 90% mutagenesis in T0 barley plants.^108^ Furthermore, heat stress and RNA-silencing suppressor are two other crucial techniques for enhancing the effectiveness of CRISPR genome engineering in plant species, including *Nicotiana benthamiana*,^109,110^ *Arabidopsis*,^111,112^ and Soybean.^113,114^ These strategies will surely aid in increasing the coherence of the CRISPR-Cas system.

## 6. Applications in Plant Breeding/Modern Agriculture

CRISPR/Cas9 is a genome-editing tool which is developing very fast, a new molecular tool and is very important for improving agriculturally important traits in various crops. A number of countries exempted genome-edited crops, which do not use transgenic DNA or any genetic material for the improvement of crops.^115^ The CRISPR/Cas 9 is a versatile tool used to improve agriculturally important crops such as quality, disease resistance, and herbicide tolerance. This technique implemented to discover oil, provide disease resistance and improve quality (Zhang et al), decrease potato browning,^116^ and mitigating volunteer rice.^117^ CRISPR/Cas9 mutagenesis in *Arabidopsis* often results in chimerism in T1 generation due to low expression of Cas9 during zygote and early embryo developmental stages.^12^ Virdi et al studied that the KASI gene of soyabean is crucial for conversion of sucrose to oil. Thus, the GmKASI gene is disrupted by reciprocal chromosomal translocation.

### 6.1. CRISPR/Cas9 as a Tool for Crop Improvement

Genome editing has introduced important agricultural traits including heat, cold, and herbicide tolerance and increased shelf life of the crop (Table 1). The fourth largest growing crop in India is potato, and it is staple food and India is the 2nd largest producer of potato in the world. The major problem growers and farmers faced is the enzymatic browning which decrease the production as well as quality of processed product. Gonzalez et al reported successful application of CRISPR for reducing the enzymatic browning in potato tuber by targeting the Polyphenol Oxidase 2 (StPPO2). By disrupting this gene, the enzymatic browning reduced by 73% and PPO activity by 69%. Volunteer rice are the rice which germinated from the seeds falling into the field during the harvest season and grow in the next spring. If volunteer rice grows from the feed variety, it compromises the quality of rice meant for human consumption. The japonica rice is resistant to beta-triketone herbicides (bTH) such as benzobicyclon (BBC), the authors tested the feasibility of engineering BBC susceptibility in japonica rice (cv. Nipponbare) by targeting the HIS1 gene using cytosine base editor (CBE). They eliminate the start codon or introduce premature stop codon within HIS1 coding sequence. The HIS1 loss-off function lines appear to be susceptible to BBC and other beta triketone pesticides, paving a way to control the germination of volunteer rice.

**Table 1.** Role of CRISPR/Cas9 gene-editing technology on different agricultural crops with enhanced or improved trait.

Crop	Enhanced or Improved trait by CRISPR	Target Gene	References
Arabidopsis thaliana	Drought resistance	AtOST2	^118^
Arabidopsis thaliana	Salt resistance	AtWRKY and AtWRKY4	^119^
Arabidopsis thaliana	Metal stress tolerance	Atoxp1	^120^
Beta vulgaris	Severe curly top virus	IR	^121^
Brassica oleracea	Mosaic virus	CP	^122^
Brassica napus	Herbicide resistance	OsALS	^17^
Capsicum frutiscens	Leaf curl virus	C1/C4 + V1/V2	^123^
Cucumis sativus	Mosaic virus	ORF1a, ORF 3a, 3 0UTR	^124^
Glycine max	Salt resistance	GmDrb2a and GmDrb2b	^125^
Gossypiun herbaceum	Leaf curl Multan virus	Rep + IR	^26^
Gossypiun herbaceum	Leaf curl virus and beta satellite	Rep	^126^
Gossypium hirsutum	Heat resistance	GhPGF and GhCLA1	^127^
Manihot esculenta	Cyanide reduction	CYP79D1 and CYP79D2	^128^
Manihot esculenta	Herbicide resistance	OsALS	^129^
Medicago sativa L.	Biomass yield and quality	Msfta1	^130^
Musa sp.	Streak virus	ORF1, 2, 3	^131^
Oryza sativa	Salt resistance	OsDST	^132^
Oryza sativa	Salt resistance	OsSPL10	^133^
Oryza sativa	Heat tolerance	OsPDS	^134^
Oryza sativa	Herbicide resistance	OsTB1	^135^
Oryza sativa	Metal stress tolerance	OsARM1	^75^
Oryza sativa	Metal stress tolerance	OsNramp5	^136^
Oryza sativa	Metal stress tolerance	OsLCT1	^137^
Oryza sativa	Metal stress tolerance	OsHAK1	^138^
Oryza sativa	Metal stress tolerance	OsPRX2	^139^
Oryza sativa	Bacterial blight	OsSWEET13	^140^
Oryza sativa	Plant hopper	OsCYP71A1	^141^
Oryza sativa	Stem borer Rice	OsCYP71A1	^141^
Phaseolus vulgaris	Yellow dwarf virus	LIR	^142^
Solanum lycopersicum	Powdery mildew	SlMLO	^143^
Solanum lycopersicum	Late blight	miR482b and miR482c	^144^
Solanum lycopersicum	Gray mould	SlPL	^145^
Solanum lycopersicum	Cold resistance	SlCBF1	^146^
Solanum lycopersicum	Bacterial leaf spot disease	SlJAZ2	^147^
Solanum lycopersicum	Yellow leaf curl virus (TYLCV)	IR	
Solanum lycopersicum	Yellow leaf curl virus	CP	^148^
Solanum tuberosum	Potato virus	P3, CI, Nib, CP	
Triticum aestivum	Dwarf virus	MP/CP + Rep/RepA + LIR	^149^

### 6.2. CRISPR/Cas9 for Abiotic Stress Tolerance

Abiotic stress is the natural condition of environment in which either there is high or low amount of natural environmental condition which affect the growth and development of plant. For example, heat, water, cold, drought etc. The stress caused due to these factors affect the growth of plant and hence reduce the plant growth. The Crispr/Cas9 genome-editing tool is very simple, accessible, and hence impart resistance against drought, salinity, heat, cold, metal, and herbicide stresses.

CRISPR/Cas9 has great potential for transforming agriculture by making plants tolerant to biotic and abiotic stresses and improving their nutritional value and yield. Acceleration of plant breeding is achieved by CRISPR/Cas as a tool and technique.

## 7. Conclusion and Future Thrust

New plant breeding techniques open avenues for researchers by facilitating the ability to precisely and quickly insert the desired traits than conventional breeding. CRISPR/Cas9-based genome editing is a fundamental breakthrough technique to accelerate plant breeding and crop improvement program of various crops. With the rapid development of CRISPR/Cas9 technology during the last 4 years, the promise of a next green revolution with new crop varieties meeting long-standing requests for better adaptability in the changing environment like photo-thermo insensitivity, biological fixation of nitrogen, biofortification, and efficient biofuel production could be realized in the near future.

In the last few years, it is being applied in many plant species for improving yield, combating biotic and abiotic stresses, multiplex editing, improving nutritional value, as well as to improve other economically important traits. Nevertheless, CRISPR/Cas9-based genome editing is a vital technique to obtain “genome edited” widely used in staple crops globally that will help to achieve the hunger and poverty free globe to feed the growing human population. Furthermore, numerous modifications to this technology are needed to increase on-target efficiency as most work carried is preliminary and needs further improvement. A major advantage of using CRISPR/Cas9-induced genome editing is to provide an opportunity for targeting multiple sites simultaneously. Novel applications of this technology is conferring multiple pathogen resistances to crop plants.

CRISPR/Cas9 has triggered innovative applications in crop improvement and accelerated breeding programs in various crops. Hence, CRISPR/Cas9 is most reliable and novel technique for transforming agriculture and opens the gateway for new advancements in the gene-editing system in plants. Various applications of genome editing tools in crop improvement to enhance crop yield, improve nutritional value, resistance to biotic and abiotic stresses, quality improvement, and other economically important traits will be a prominent area of work in the near future.
